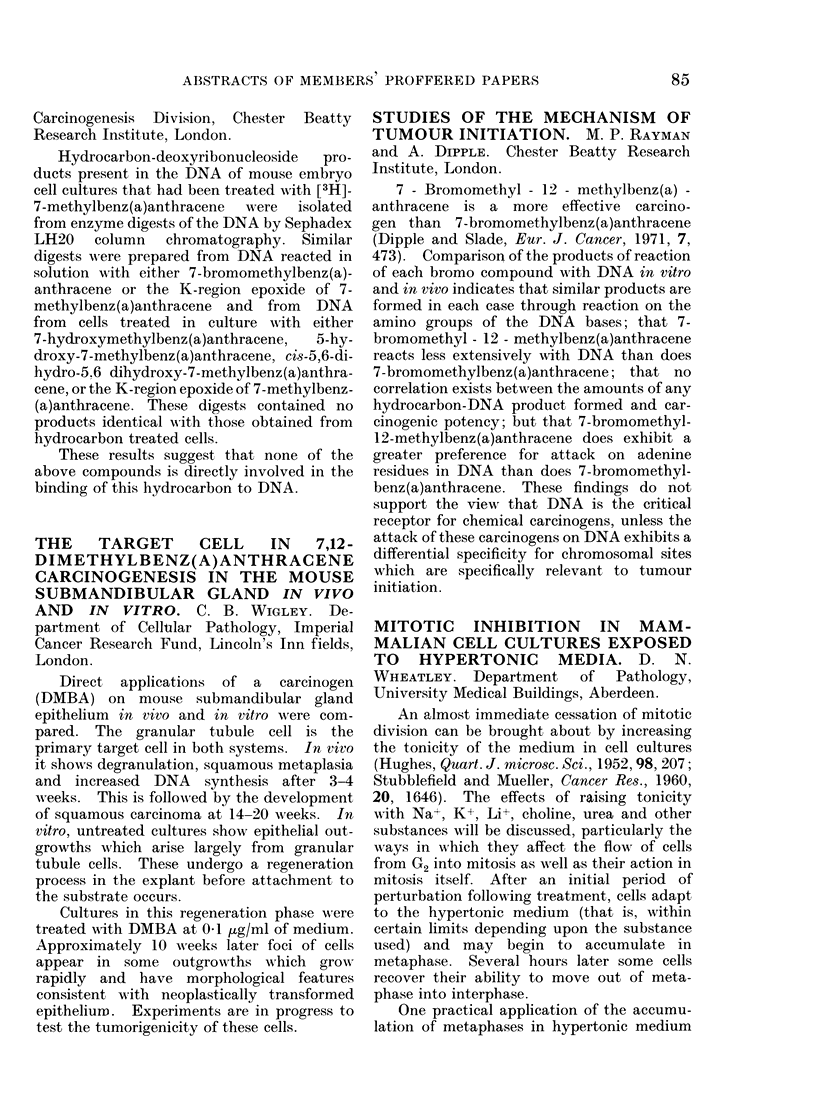# Studies of the mechanism of tumour initiation.

**DOI:** 10.1038/bjc.1973.105

**Published:** 1973-07

**Authors:** M. P. Rayman, A. Dipple


**STUDIES OF THE MECHANISM OF TUMOUR INITIATION.** M. P. RAYMAN and A. DIPPLE. Chester Beatty Research Institute, London.

7 - Bromomethyl - 12 - methylbenz(a) - anthracene is a more effective carcinogen than 7-bromomethylbenz(a)anthracene (Dipple and Slade, *Eur. J. Cancer*, 1971, **7**, 473). Comparison of the products of reaction of each bromo compound with DNA *in vitro* and *in vivo* indicates that similar products are formed in each case through reaction on the amino groups of the DNA bases; that 7-bromomethyl - 12 - methylbenz(a)anthracene reacts less extensively with DNA than does 7-bromomethylbenz(a)anthracene; that no correlation exists between the amounts of any hydrocarbon-DNA product formed and carcinogenic potency; but that 7-bromomethyl-12-methylbenz(a)anthracene does exhibit a greater preference for attack on adenine residues in DNA than does 7-bromomethyl-benz(a)anthracene. These findings do not support the view that DNA is the critical receptor for chemical carcinogens, unless the attack of these carcinogens on DNA exhibits a differential specificity for chromosomal sites which are specifically relevant to tumour initiation.